# CT Ventricular Volume Reference Percentile Curves Using Automated nnU-Net Segmentation: A Study with LLM-Facilitated Cohort Selection

**DOI:** 10.3390/diagnostics16203417

**Published:** 2026-10-20

**Authors:** Nathan Vishwanathan, Jakob Wasserthal, Shan Yang, Martin Segeroth, Joshy Cyriac, Johanna Maria Lieb, Michael Bach, Marios Nikos Psychogios, Matthias Anthony Mutke

**Affiliations:** Clinic of Radiology and Nuclear Medicine, University Hospital Basel, 4031 Basel, Switzerland

**Keywords:** intracranial ventricles, ventricular volume, computed tomography, ventricular nomograms, large language models, nnU-Net

## Abstract

**Background:** Age- and sex-specific CT reference percentile curves of ventricular volume may support objective assessment of ventricular enlargement. We aimed to generate such curves by combining a locally deployed large language model (LLM) for cohort curation with nnU-Net 12-subcompartment segmentation. **Methods:** This retrospective study did not require formal ethics approval under the Swiss Human Research Act. We screened 42,211 head CT reports from January 2019 to April 2024. A phrase prefilter identified 9622 candidates for German-tuned 70-billion-parameter LLM review of stroke, leukoencephalopathy, parenchymal defects, enlarged ventricles, and space-occupying lesions; 6516 remained. A second LLM workflow screened 210,761 clinical data warehouse summaries to exclude dementia, organic brain disease, or cognitive decline. After image-availability and series-mismatch exclusions, the final cohort comprised 2608 CTs from 2608 patients (1374 males, 1234 females; age 14–98 years). Ventricular masks were obtained using a validated nnU-Net tool integrated in TotalSegmentator. **Results:** LLM labelling accuracy in 471 blinded reports ranged from 0.966 to 0.992. CDW screening audit showed 91% agreement, sensitivity 0.96, and negative predictive value 0.96. Median total ventricular volume rose from 10.94 mL in young females and 13.42 mL in young males to 21.97 mL and 31.97 mL, respectively, in the 60–69 age group. Except for the fourth ventricle, compartments showed positive age correlations (*r* = 0.44–0.69); the fourth ventricle showed no relevant association (*r* = 0.01 males; *r* = 0.05 females). After adjustment for age, male sex remained associated with greater total ventricular volume (6.54 mL; 95% CI, 5.61–7.46; p<0.001). **Conclusions:** This study provides age- and sex-specific CT ventricular nomograms in a selected reference cohort. The LLM-assisted curation and nnU-Net segmentation workflow provides a feasible approach for large-scale cohort analysis.

## 1. Introduction

Accurate assessment of intracranial ventricular size is essential in the daily workflow of radiologists, playing a critical role in identifying hydrocephalus [1,2] and ex vacuo dilation due to cerebral atrophy in neurodegenerative diseases [3,4,5]. It remains challenging, as assessment is often subjective and relies heavily on the radiologist’s experience, resulting in variability [6,7].

Several methods can be used to measure ventricular size; however, volumetric measurements have proven superior to semi-quantitative 2D measurements such as the Evans Index in detecting subtle changes in ventricular size and correlating with clinical presentation [8,9,10]. This is particularly valuable in conditions such as hydrocephalus, where accurate measurement is important for monitoring treatment response.

Despite promising results, automated segmentation for ventricular volume assessment was previously considered impractical for diagnostic use because of long processing times [11]. In contrast, AI and modern machine learning algorithms provide fast, precise, and highly reproducible measurements, making automated segmentation practical for routine clinical workflows [12,13]. Previous studies on baseline ventricular volumetric measurements have been limited by either small sample sizes or less detailed subcompartment measurements [9,14,15,16].

Furthermore, large CT-based reference cohorts combining detailed ventricular subcompartment measurements with systematic radiology-report and clinical-record screening remain limited.

While both CT and MRI are widely employed in clinical neuroimaging, MRI offers superior soft tissue contrast, whereas CT is more commonly used because of greater availability, lower resource demands, and faster imaging speed, making it particularly valuable in emergency settings such as trauma and stroke [17,18,19].

To establish a clinical CT reference cohort for ventricular-volume distributions, we screened radiology reports and clinical summaries to exclude pathologies. Given the increasing role of AI in medical analytics [20,21,22,23], we used an open-source LLM to streamline this task while running it locally within our hospital’s technical infrastructure to ensure data protection compliance and avoid reliance on external resources. Selected cases were then processed using an in-house developed nnU-Net-based segmentation algorithm.

This study aimed to establish age- and sex-adjusted CT-based ventricular nomograms using an AI-driven workflow to derive a selected clinical reference cohort based on radiology-report and clinical-record screening, incorporating an in-house deployed LLM for analysing radiology reports and clinical data, followed by an nnU-Net algorithm for automated segmentation of intracranial ventricles.

## 2. Materials and Methods

### 2.1. Study Design

This retrospective study was conducted in the Department of Radiology at the University Hospital Basel. The study aimed to establish age- and sex-specific nomograms of intracranial ventricular volumes using an AI-driven workflow (Figure 1).

### 2.2. Data Acquisition

All CT examinations included in the final dataset were performed at our radiology department between 1 January 2019 and 5 April 2024. All examinations were routine non-contrast head CT studies acquired in clinical practice. Acquisition and reconstruction parameters were not standardized and varied according to scanner, protocol, and clinical indication, reflecting the real-world nature of the cohort. Scanner model and reconstructed slice-thickness distributions are provided in Supplementary Figure S11.

### 2.3. Exporting and Preprocessing Relevant Data

We searched for head CTs in our hospital database using an in-house developed PACS search tool (PACS Crawler, Department of Radiology and Nuclear Medicine, University Hospital Basel, Basel, Switzerland). The search initially identified 181,965 examinations, from which 42,211 head CT examinations with available radiology reports were selected for further screening. Available text-based exam data, including patient ID, age, sex, exam date, exam identifier, and radiology reports including medical history, were extracted and anonymized.

A Python script (Python version 3.12) further filtered the dataset using specific report phrases: “no intracranial bleeding,” “no midline-shift,” and “no atrophy” for patients under 60 years of age, and “no atrophy,” “low atrophy,” or “minimal atrophy” for patients aged 60 years or older. This report-based prefilter served as a pragmatic surrogate for the absence of major intracranial pathology or more-than-mild age-related atrophy and reduced the 42,211 examinations to 9622 candidate examinations for LLM review.

### 2.4. LLM-Based Screening of Radiology Reports

Radiology reports were analysed using the open-source LLM “llama3-sauerkraut:70B-Q5-K-M” [24] based on Meta-Llama-3-70B-Instruct (Meta Platforms Inc., Menlo Park, CA, USA) [25], chosen because it was fine-tuned for German language. Processing was facilitated using Ollama (ver. 0.2.5) [26], allowing local hardware processing to ensure data security. Neither the platform nor the model was modified for this study. To ensure deterministic outputs, temperature was set to 0.0 [27].

Because the radiology reports followed a uniform structure, we extracted the neurocranium and conclusion sections to ensure relevance, avoid input overflow, and improve processing time. The LLM evaluated five key pathologies: stroke, parenchymal defects, space-occupying lesions, enlarged ventricles, and white matter lesions (WML) beyond Fazekas 1. A detailed overview of the radiology-report prompts is provided in Supplementary Table S6. The LLM responded dichotomously (“yes” or “no/not mentioned”) and provided a short explanation supporting the decision.

Out of 9622 radiology reports, 5805 cases showed no significant findings in the examined categories. An additional 711 cases with isolated WML and no other pathology were also included, yielding 6516 cases for the next step. To validate report screening, 471 randomly selected radiology reports with blinded LLM results were manually reviewed by a medical doctor, and the findings were compared with the LLM output.

### 2.5. LLM Analysis of Clinical Data

Of the 6516 cases, 1492 cases from 1483 unique patients lacked clinical data in our Clinical Data Warehouse (CDW); 1431 of these were teleradiology cases from external hospitals. The remaining 5024 cases corresponded to 210,761 clinical summaries, comprising all records available in the electronic health record for each patient.

To exclude patients with conditions that could bias ventricular nomograms, CDW clinical summaries were screened in two steps. First, a concise single-question prompt was used to reduce the size of the dataset and exclude summaries with low direct relevance. Each medical summary was processed individually by the LLM, and 17,932 summaries were identified for further analysis. Second, a more specific prompt screened for dementia or mild cognitive impairment, structural brain disease, and cognitive decline. A positive response to any of these questions led to exclusion of the patient and all corresponding CT examinations. Prompt wordings are provided in Supplementary Tables S7 and S8. In total, 7037 summaries yielded a positive finding, leading to exclusion of 1637 patients and 1852 studies.

Given the scale of the CDW dataset, a pragmatic sample of 100 clinical summaries was selected for manual quality-control assessment, balancing feasibility of manual review with coverage of the different workflow outcomes. The sample was stratified to include 25 summaries classified as negative after the first LLM step, 25 classified as negative after the second step, and 50 classified as positive after the second step. Manual review was performed by a radiology resident using the same exclusion-relevant categories as in the CDW workflow.

After CDW screening, 3172 examinations from 3040 unique patients remained. Eighty-six examinations from 76 unique patients were excluded because of PACS retrieval errors (see Figure 1). The remaining 3086 examinations from 2964 unique patients were retrieved. To avoid repeated measurements from the same individual, only the earliest eligible CT examination per patient was retained, resulting in 2964 examinations. All retrieved datasets were then manually reviewed at the series level for suitability for ventricular volumetry. Examinations were excluded when the retrieved dataset did not contain the intended native head CT acquisition and instead consisted of angiographic, venographic, CAS, or otherwise mismatched/non-native series. This refinement excluded 356 examinations, resulting in a final reference cohort of 2608 CT examinations from 2608 patients.

### 2.6. Automated Ventricular Segmentation and Volumetry

The selected cases were processed with a previously developed and validated in-house deep-learning algorithm trained to segment the intracranial ventricles and in total 12 anatomical subcompartments. The algorithm is integrated into the TotalSegmentator framework [28] and was systematically evaluated in a separate validation study [29]. In prior validation, the model demonstrated a median Dice coefficient of 0.91 and a mean Dice coefficient of 0.83. In the present study, we deployed the previously trained model without additional retraining. A representative ventricular segmentation and its corresponding 3D reconstruction are shown in Figure 2 and Figure 3, respectively.

### 2.7. Segmentation Plausibility Check

As the segmentation model had already been technically validated in an independent study, the visual review performed here was intended as a plausibility check to identify major segmentation failures after application of the previously validated model to the study cohort, rather than as a repeat validation of segmentation accuracy. A random subset of 120 examinations and their corresponding segmentation masks was visually reviewed by a radiology resident for major segmentation failures and visually apparent over- or undersegmentation. No visually apparent major segmentation failures were identified. Selected examples of minor segmentation variance are shown in Figure 4.

### 2.8. Statistical Analysis

Descriptive statistics included means, standard deviations, medians, interquartile ranges, percentile estimates, and 95% confidence intervals. LLM validation performance was summarized by sensitivity, specificity, accuracy, positive predictive value, and negative predictive value. Age-volume associations were assessed using Pearson correlation coefficients stratified by sex. Because the age-volume relationship was not assumed to be strictly linear, Spearman rank correlations were additionally calculated for robustness. Linear regression was used to estimate age-related changes in total ventricular volume separately by sex. A multivariable linear regression model including age and sex was additionally used to assess sex differences in total ventricular volume after adjustment for age.

For the age- and sex-specific ventricular volume nomograms, patients were stratified by sex and grouped into 10-year age bins. Ten-year age bins were chosen to provide clinically interpretable age groups while avoiding excessive fragmentation of the cohort. Within each sex- and age-bin group, empirical 5th, 10th, 15th, 25th, 40th, 50th, 60th, 75th, 85th, 90th, and 95th percentile estimates were calculated for total ventricular volume, lateral ventricular volumes, and all ventricular subcompartments. Decade-wise percentile estimates were smoothed across age groups for visualization. In addition, 95% confidence intervals for the median volume estimates were calculated for each age group and ventricular parameter using nonparametric bootstrap resampling with 1000 iterations.

All statistical analyses were performed using Python 3.12 with pandas 2.2.3, NumPy 2.1.1, SciPy 1.14.1, and Matplotlib 3.9.2.

## 3. Results

### 3.1. LLM Analysis of Radiology Reports

Out of 9622 radiology reports, the LLM identified positive cases in the following categories: stroke (*n* = 1532), parenchymal defects (*n* = 1880), space-occupying lesions (*n* = 679), enlarged ventricles (*n* = 1702), and isolated WML beyond Fazekas 1 without other LLM-flagged pathology (*n* = 711).

A total of 471 cases were manually reviewed and used as ground truth. The LLM demonstrated high accuracy across categories: 0.975 for stroke, 0.966 for parenchymal defects, 0.968 for space-occupying lesions, 0.992 for enlarged ventricles, and 0.979 for WML beyond Fazekas 1. Detailed metrics are provided in Table 1. Q3 and Q5 were internal pipeline labels corresponding to criteria already covered by the preceding Python-based phrase prefilter. Because they were redundant with the pre-screening step, they were not retained as separate outcome categories in the final validation analysis. Exact 95% confidence intervals for sensitivity, specificity, PPV, and NPV are provided in Supplementary Table S5.

### 3.2. LLM Analysis of Clinical Summaries

Out of 210,761 clinical summaries, the LLM flagged 17,932 for further processing using the single-question triage prompt. These were then evaluated with the three-question prompt, resulting in positive findings for dementia/MCI (n = 861), structural/organic brain diagnoses (n = 3372), and explicit mentions of cognitive impairment (n = 4209). The respective patients were excluded on this basis.

In an additional stratified manual audit of the CDW screening workflow, 100 clinical summaries were manually reviewed: 25 classified as negative after the first LLM step, 25 classified as negative after the second LLM step, and 50 classified as positive after the second LLM step. Overall agreement between manual review and LLM workflow classification was 91% (91/100; 95% CI, 83.8–95.2). Using manual review as the reference standard, sensitivity, specificity, positive predictive value, and negative predictive value were 0.96 (43/45; 95% CI, 0.85–0.99), 0.87 (48/55; 95% CI, 0.76–0.94), 0.86 (43/50; 95% CI, 0.74–0.93), and 0.96 (48/50; 95% CI, 0.87–0.99), respectively. Agreement within the three audit groups was 92% (23/25), 100% (25/25), and 86% (43/50), respectively.

### 3.3. Cohort Details

The final cohort comprised 2608 examinations from 2608 patients (1374 male, 1234 female). Male age was 14–97 years (mean ± SD 45.73 ± 18.49; median 44 years, IQR 30–60), and female age was 16–98 years (mean ± SD 53.46 ± 19.10; median 54 years, IQR 38–69). The age structure of the cohort is detailed in Figure 5.

### 3.4. Ventricle Measurements

In the youngest decade (10–19 years; mean age 17.96 years in females and 17.93 years in males), the median total ventricular volume (TVV) was 10.94 mL (IQR 7.88) in females and 13.42 mL (IQR 9.27) in males. TVV was defined as the sum of the left and right lateral ventricle volumes and the third and fourth ventricles. Median TVV increased to 17.08 mL (IQR 10.96) in women and 20.61 mL (IQR 11.44) in men in the 50–59 age group, and to 21.97 mL (IQR 13.58) and 31.97 mL (IQR 17.33), respectively, in the 60–69 age group. Age- and sex-specific median ventricular volumes and interquartile ranges are shown in Figure 6.

At the subcompartment level, the same decade step was observed across most lateral subcompartments and the third ventricle. Comprehensive decade-stratified means and standard deviations for all 12 subcompartments are reported in Supplementary Tables S1 and S2. Detailed percentile reference values are provided in Supplementary Table S3 (female) and Table S4 (male), and visualized as age–percentile curves in Supplementary Figure S1.

### 3.5. Age-Related Correlation of Ventricular Volumes

Except for the fourth ventricle, all compartments showed moderate to strong positive correlations with age (*r* = 0.44–0.69). The third ventricle exhibited the steepest age-related expansion. The fourth ventricle showed only minimal association with age (*r* = 0.01 in males; *r* = 0.05 in females; Figure 7). Spearman rank correlation analysis yielded the same overall pattern of age-related associations, supporting the observed age-volume relationships. The Spearman rank correlations are provided in Supplementary Figure S10.

Linear regression showed increasing total ventricular volume with age in both females (0.48 mL/year; 95% CI, 0.44–0.51; p<0.001) and males (0.52 mL/year; 95% CI, 0.48–0.56; p<0.001). After adjustment for age, male sex remained associated with greater total ventricular volume (6.54 mL; 95% CI, 5.61–7.46; p<0.001).

## 4. Discussion

In this study, we used a multi-step AI pipeline consisting of an in-house deployed open-source LLM to flag radiology reports according to predefined criteria and to analyse clinical data for cohort establishment, followed by an in-house nnU-Net-based algorithm to produce sex- and age-adjusted CT ventricular nomograms across 12 subcompartments. Previous studies were limited by smaller cohorts or less detailed ventricular measurements and did not utilise modern AI tools for systematic large-scale cohort establishment [9,14,15,16].

We demonstrated good LLM performance via a blinded audit of 471 reports, with accuracies between 0.966 and 0.992 across target categories. The additional summary-level audit of the CDW screening step further supported the exclusion-oriented workflow, showing high sensitivity and negative predictive value. Although sensitivity for space-occupying lesions was lower than for the other categories, only 4 of 471 manually reviewed reports represented false-negative classifications (0.85%), corresponding to a low absolute rate of missed SOL findings in the validation sample. Potential weaknesses such as hallucination, imprecision on ambiguous phrasing, and failures with chaotic or overlong inputs were addressed by prompt engineering, deterministic decoding, schema-constrained outputs, and short justifications for each YES/NO decision. We further split the workflow into substeps and trimmed inputs to avoid context overflow, which improved stability at scale.

Using LLMs substantially reduced the amount of manual review required for cohort selection while achieving high accuracy in labelling. This was also shown by Beattie et al. [31], who used commercial LLMs to automate clinical trial matching with high accuracy. In contrast, our analysis relied on an open-source model, enabling local processing without reliance on external commercial systems.

Among subcompartments, most lateral ventricular subdivisions showed moderate to strong positive correlations with age, with the third ventricle exhibiting the steepest increase, whereas the fourth ventricle showed no meaningful association, consistent with previous studies [32,33]. Across compartments, male ventricular volumes exceeded female volumes. For total ventricular volume, this difference persisted after adjustment for age. However, intracranial volume was not available for adjustment, and sex-related differences should therefore be interpreted cautiously.

Given the smaller size of some ventricular subcompartments, such as the temporal or frontal horns, measuring the ventricles as a whole does not capture regional changes. This is relevant because ventricular alterations can exhibit disease-specific patterns due to atrophy in nearby structures. In Alzheimer’s disease, for example, temporal horn widening linked to hippocampal atrophy has been recognized as a marker correlating with disease progression [34]. Measuring such regional features separately and comparing the values to baseline may therefore help identify subtle changes [35].

The visual plausibility check of the segmentations was conducted by a single radiology resident and was intended to identify major segmentation failures in a random sample after applying the previously validated segmentation algorithm to the study cohort, rather than to serve as a new validation study [29]. The use of different CT scanner models and reconstructed slice thicknesses reflects routine clinical imaging and may have introduced additional variability in ventricular volume measurements.

The limitation of our cohort lies in the smaller number of older subjects, particularly among males above 90 years of age, resulting in wider confidence intervals and less reliable percentile estimates in the oldest age groups. Our deliberate exclusion strategy reduced the dataset from 42,211 head CTs with structured reports to 2608 final CT examinations, which accounts for the smaller sample sizes in the oldest decades. The age-dependent allowance of minimal or low atrophy from 60 years onward was a pragmatic selection criterion, consistent with previous literature showing increasing age-related brain volume loss in later adulthood [36]. This criterion may nevertheless have influenced ventricular-volume distributions. Conversely, requiring complete absence of reported atrophy across all ages could preferentially select unusually preserved older individuals and introduce an additional selection bias.

The provided nomograms represent reference distributions for a selected clinical head CT population aged 14 years and older at our centre, selected by radiology-report and clinical-record screening for the absence of predefined exclusion-relevant findings, and are not intended as healthy-volunteer normative values. Intracranial volume was not available in this retrospective analysis; therefore, sex-related differences could not be adjusted for head size. External validation in independent cohorts would be valuable, but is limited by the lack of public datasets combining native head CT imaging with structured radiology reports and longitudinal CDW/EHR summaries. The present work should therefore be interpreted as a single-centre reference-cohort study and proof of concept for deriving CT ventricular nomograms from routinely acquired clinical data using local AI-assisted cohort curation and automated segmentation.

## 5. Conclusions

This study integrates a locally deployed LLM for large-scale cohort selection with a nnU-Net segmentation model, providing age- and sex-specific CT ventricular nomograms across 12 subcompartments. The pipeline demonstrated robust performance in large-scale data analysis. Ventricular nomograms that include detailed subdivision measurements could help detect region-specific enlargement patterns tied to pathologies such as Alzheimer’s disease. Measured volumes align generally with existing CT/MRI studies, although direct comparison is complicated by differences in imaging protocols and cohort composition.

## Figures and Tables

**Figure 1:**
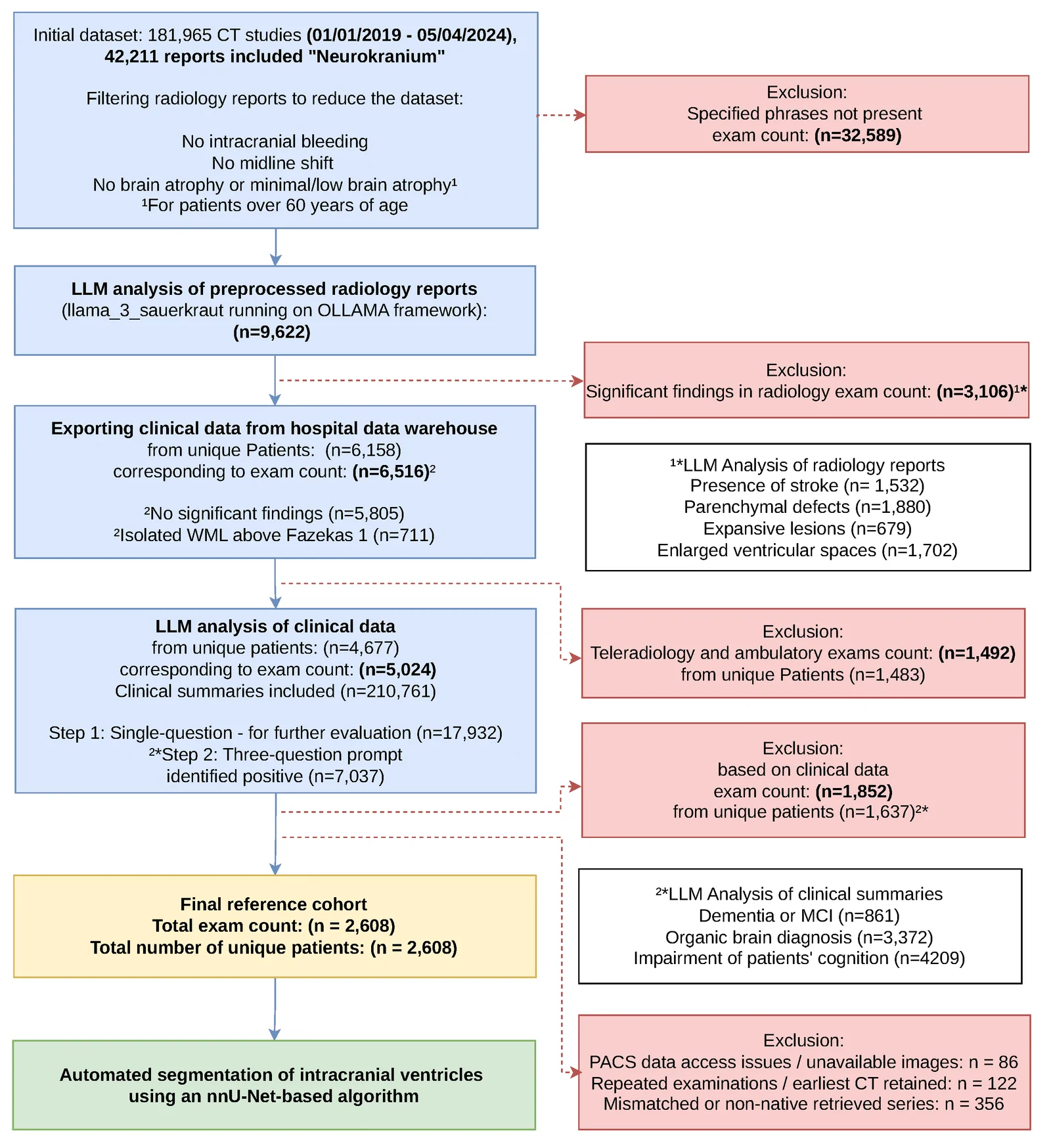
Flowchart illustrating the AI-driven workflow integrating LLM analysis and nnU-Net segmentation. Superscript ^1^ denotes a rule applied to patients aged 60 years or older, and ^2^ denotes cases with no significant findings in radiology reports or isolated WML above Fazekas 1. Superscripts ^1^* and ^2^* indicate exclusions based on LLM analysis of radiology reports and clinical summaries, respectively.

**Figure 2:**
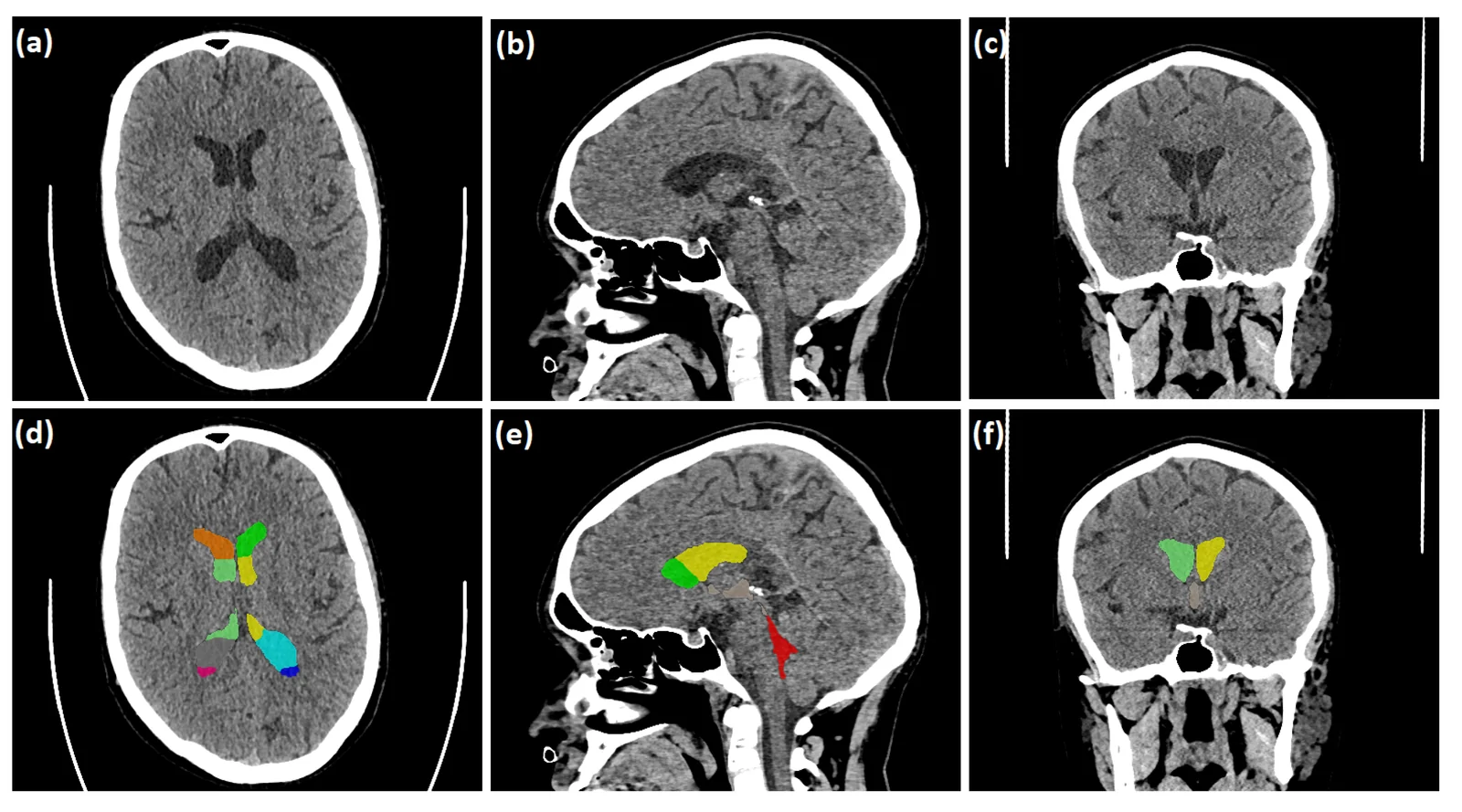
CT imaging of a 26-year-old male patient, showing axial (**a**), sagittal (**b**), and coronal (**c**) slices, and their respective ventricle segmentation masks (**d**–**f**).

**Figure 3:**
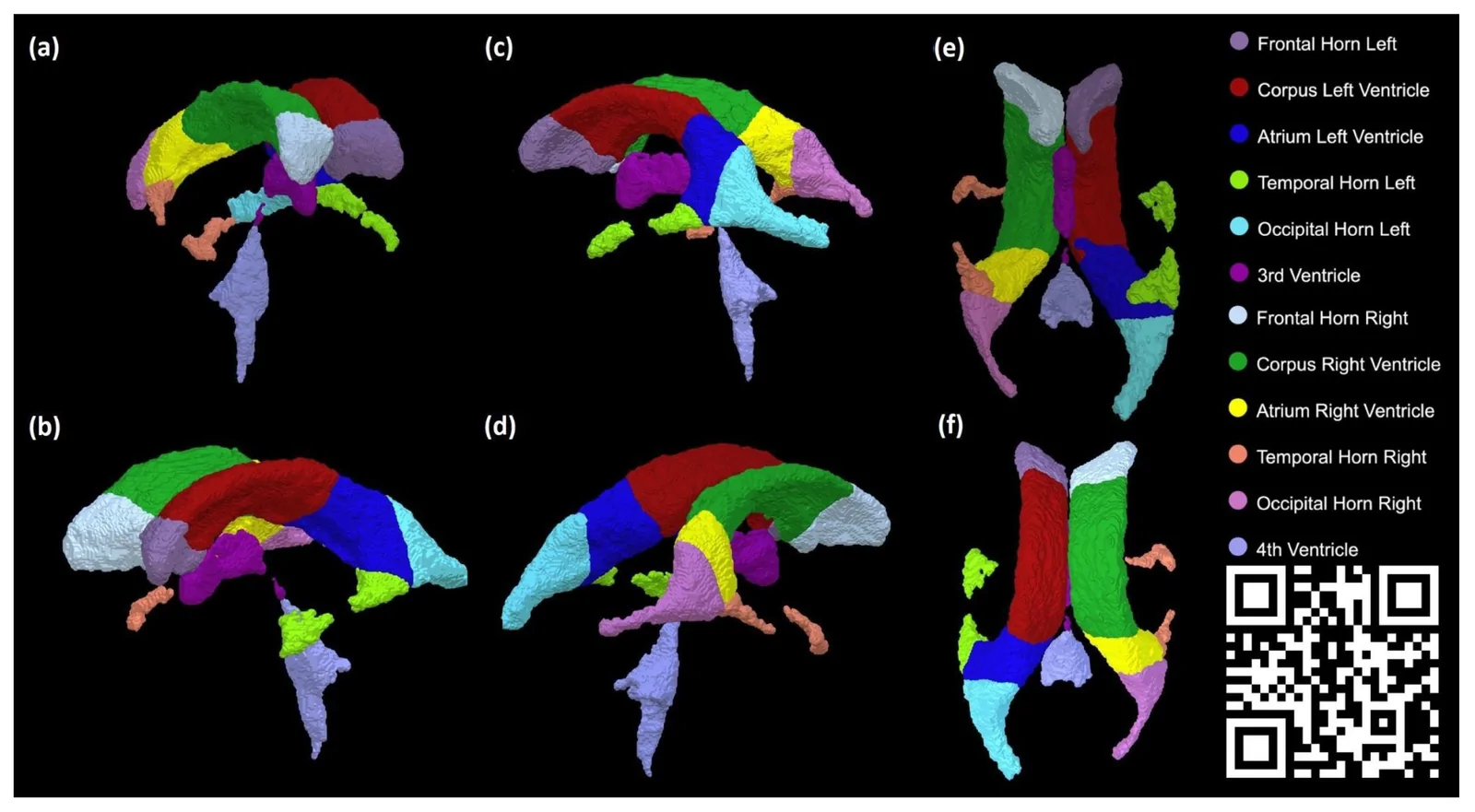
3D-rendered reconstruction of segmentation masks illustrated in Figure 2 in the same 26-year-old male patient. The NIfTI ventricle segmentation masks were converted to a 3D object (.obj file) and visualized using Microsoft 3D Viewer [30]. The views captured are: anterolateral right (**a**), anterolateral left (**b**), posterolateral left (**c**), posterolateral right (**d**), inferior (**e**), and superior (**f**). (QR code links to the online interactive model.)

**Figure 4:**
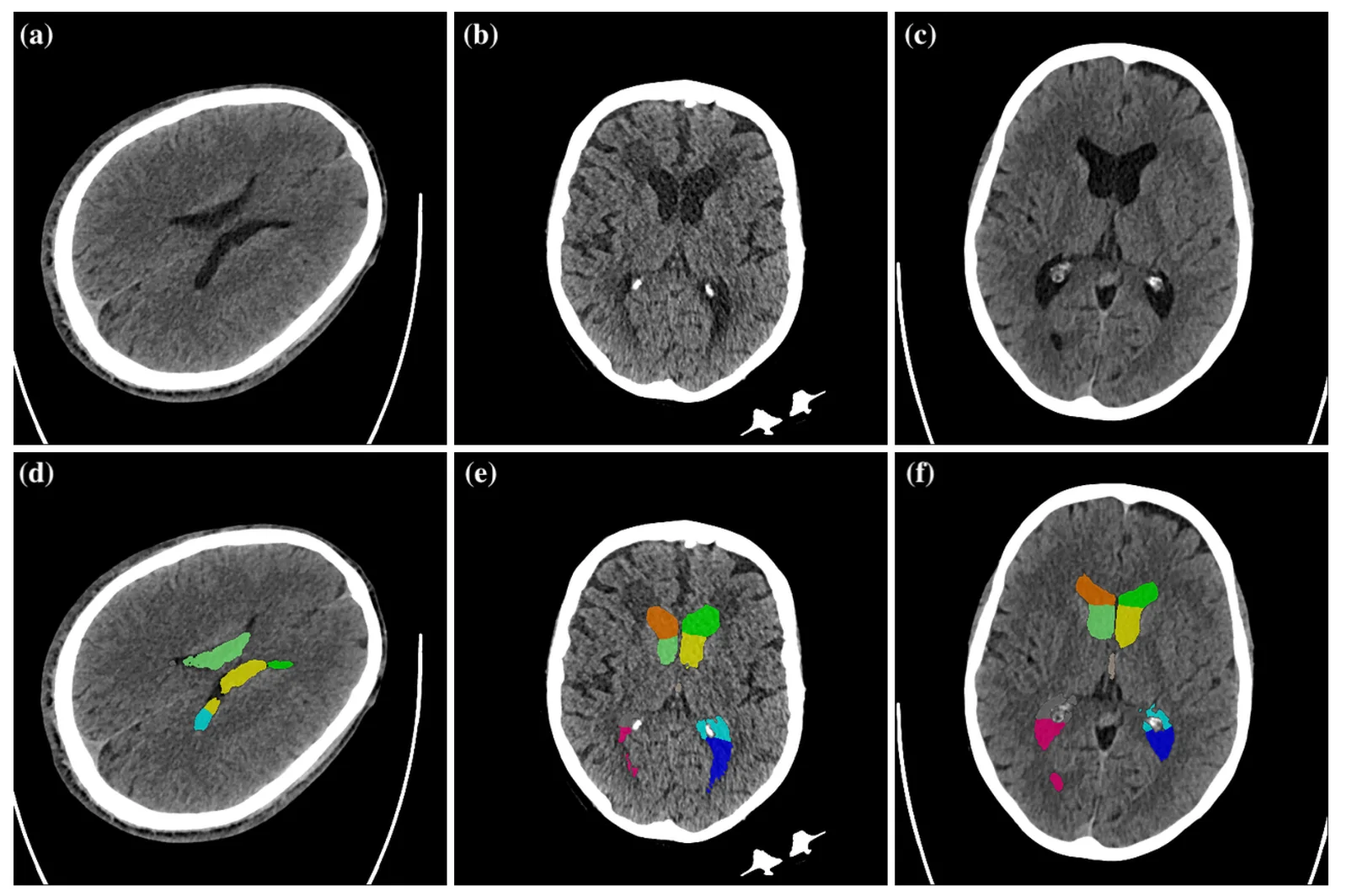
Examples from the post-deployment segmentation plausibility check illustrating minor segmentation variance. Panels (**a**–**c**) show native axial CT images, and panels (**d**–**f**) show the corresponding segmentation overlays. Panels (**a**,**d**) depict non-standard head positioning associated with mildly imprecise contouring of the lateral ventricles. Panels (**b**,**e**) show slight oversegmentation of the ventricles, particularly at the frontal horns, in the presence of periventricular leukoencephalopathy. Panels (**c**,**f**) demonstrate partial undersegmentation of the inferior portion of the third ventricle. This case also includes a partially collapsed occipital horn; the ventricular portion posterior to the collapse was nevertheless correctly recognized and assigned to the occipital horn compartment. Colors indicate individual ventricular subcompartments.

**Figure 5:**
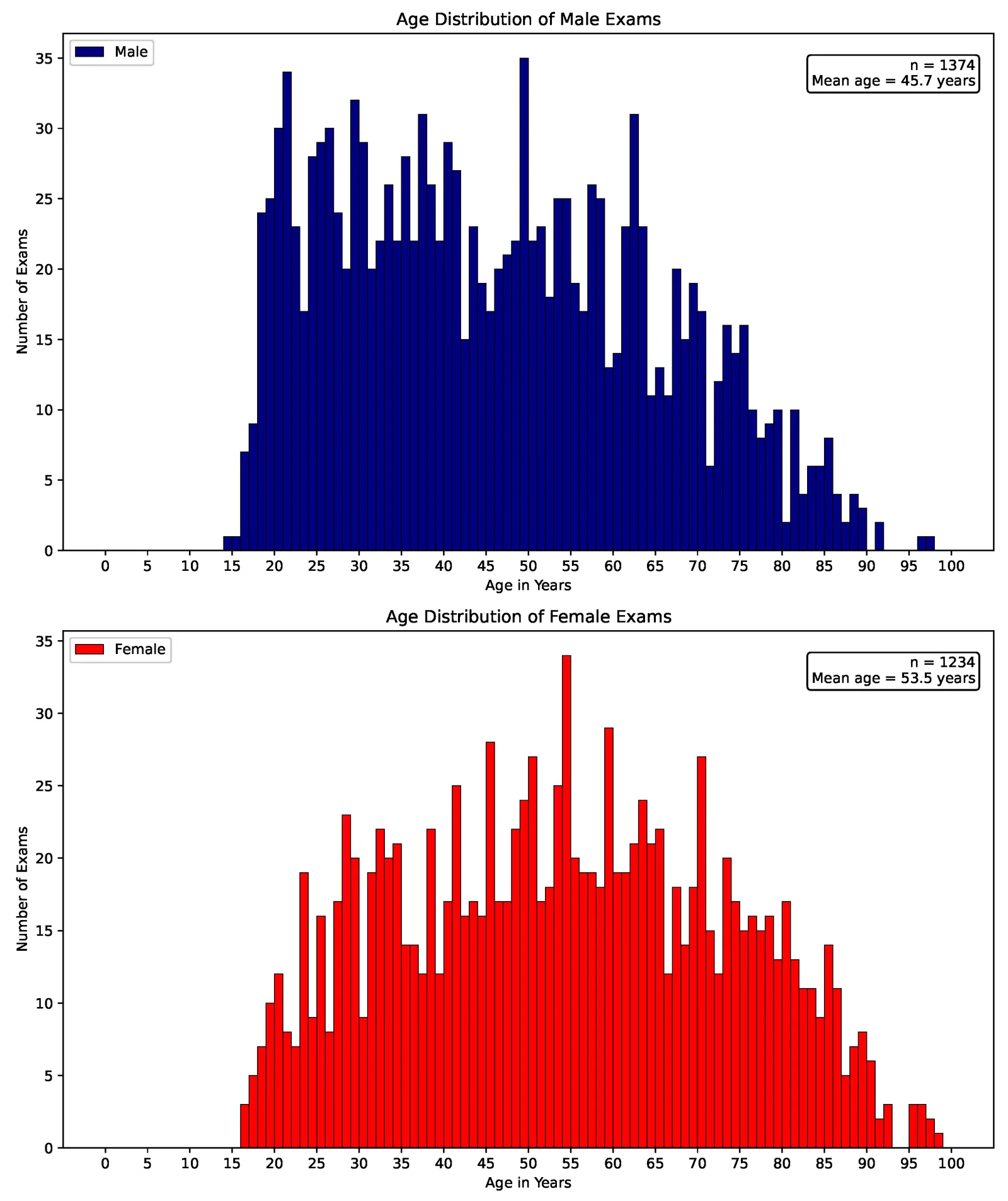
Age distribution of the final cohort by sex. Histograms show age at CT examination for males (**upper** panel, blue) and females (**lower** panel, red). The x-axis indicates age in years, and the y-axis indicates the number of CT examinations. Insets show cohort size and mean age.

**Figure 6:**
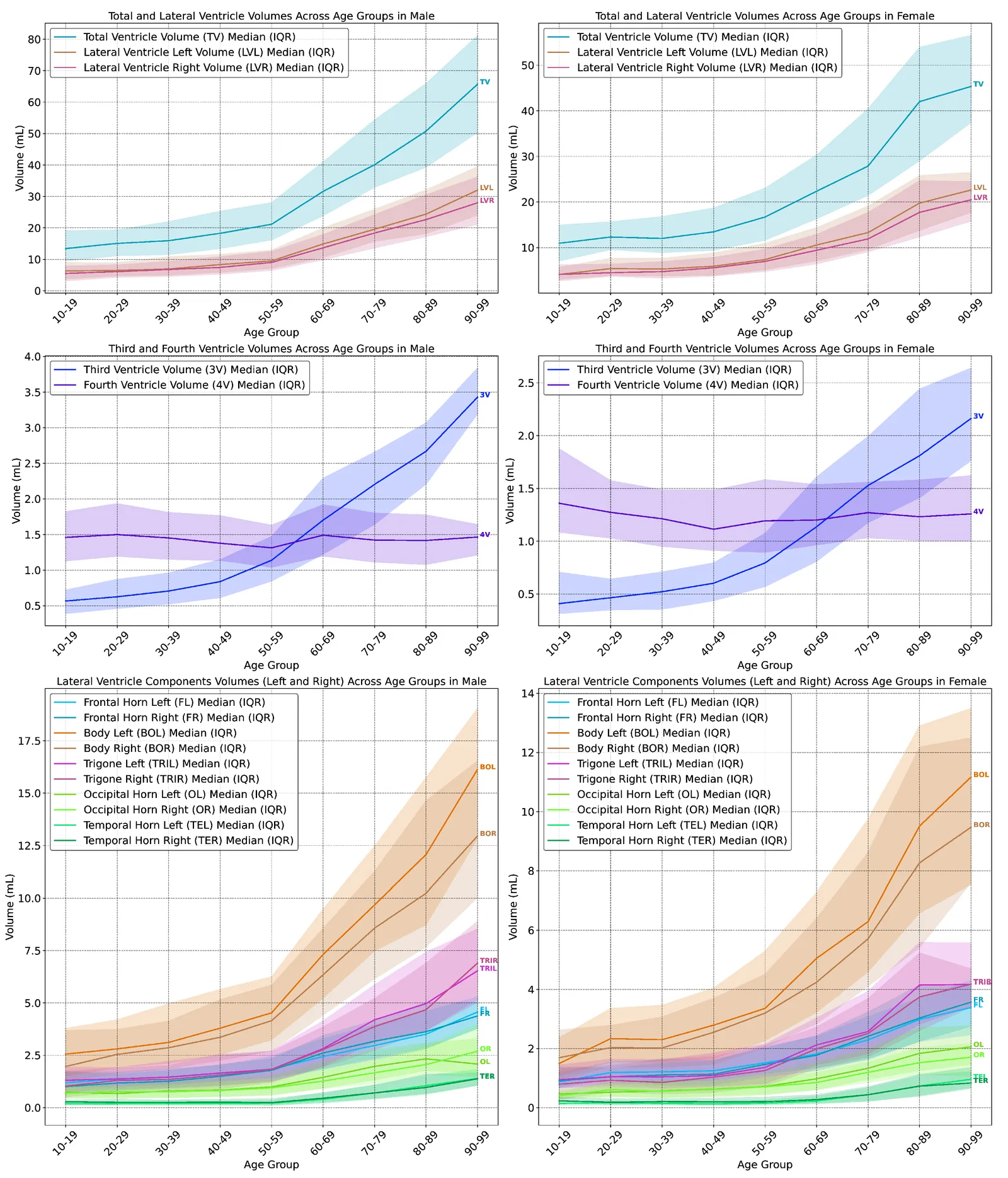
Median curves show ventricular volumes across age decades for males (**left**) and females (**right**). Shaded bands indicate the interquartile range (IQR). Panels show total and lateral ventricular volumes, third and fourth ventricular volumes, and lateral ventricular subcompartments. Volumes are reported in millilitres (mL).

**Figure 7:**
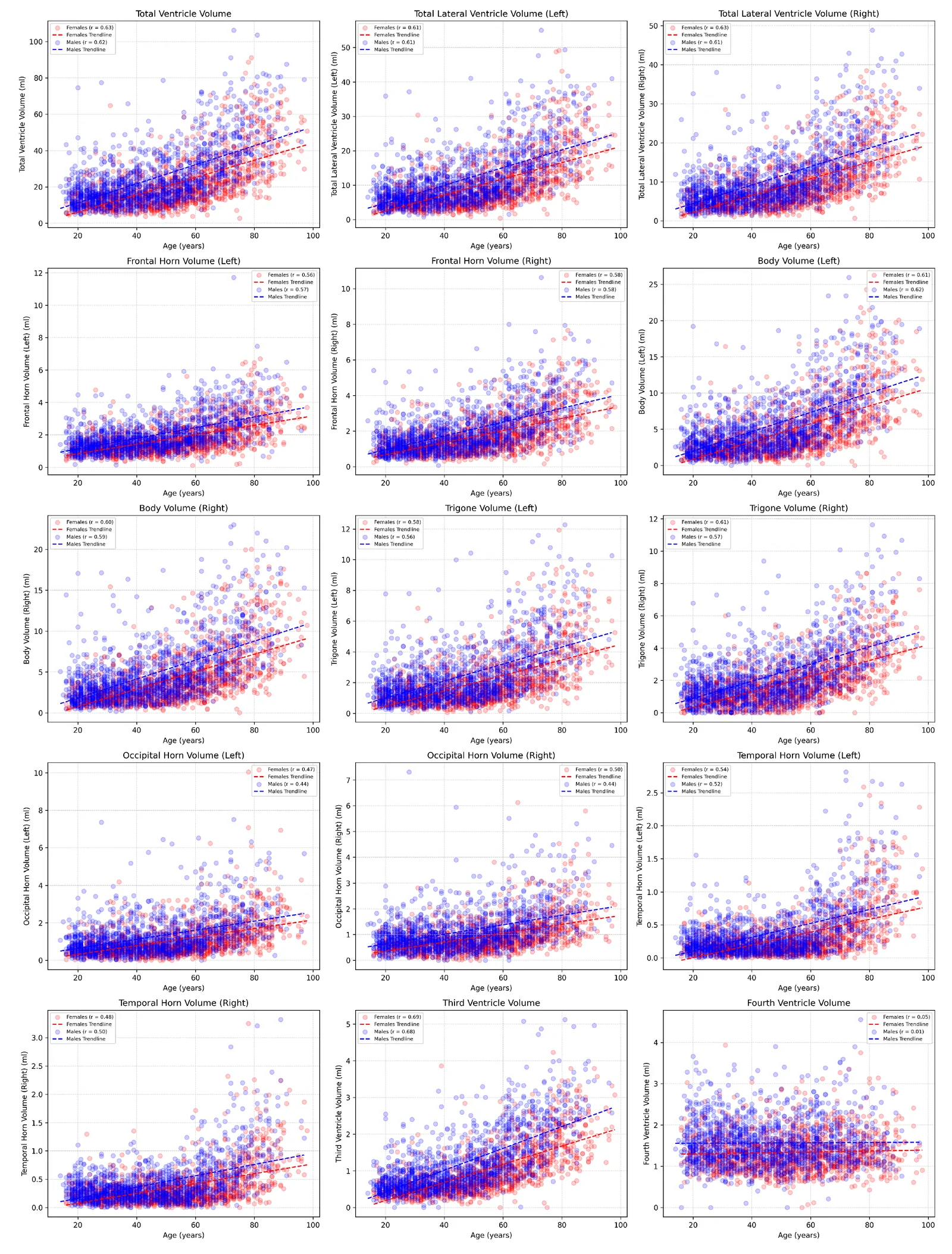
Scatter plots show ventricular volumes in relation to age. Each point represents one CT examination. Females are shown in red and males in blue; dashed lines indicate sex-specific linear trend lines. Pearson correlation coefficients are shown in each panel.

**Table 1:** LLM Performance in Manually Reviewed Cases.

Question	TP	TN	FP	FN	Sensitivity	Specificity	Accuracy	PPV	NPV	Prevalence	F1
Q1 (Stroke)	53	406	7	5	0.914	0.983	0.975	0.883	0.988	0.123	0.898
Q2 (Parenchymal defects)	73	382	4	12	0.859	0.990	0.966	0.948	0.970	0.180	0.901
Q4 (SOL)	18	438	11	4	0.818	0.976	0.968	0.621	0.991	0.047	0.706
Q6 (Enlarged ventricles)	55	412	2	2	0.965	0.995	0.992	0.965	0.995	0.121	0.965
Q7 (WML beyond Fazekas 1)	69	392	10	0	1.000	0.975	0.979	0.873	1.000	0.146	0.932

TP = True Positives, TN = True Negatives, FP = False Positives, FN = False Negatives, SOL = space-occupying lesion, F1 = F1 Score.

## Data Availability

The supplementary figures, tables, prompts, and analysis scripts associated with this study are available at Zenodo (Version v5): https://zenodo.org/records/23286133, (accessed on 14 October 2026) Due to institutional, legal, and data-protection restrictions, the underlying patient-level clinical and imaging data are not publicly available.

## Supplementary Materials

Supporting information is available at Zenodo (version v5): https://zenodo.org/records/23286133 (accessed on 14 October 2026). **Supplementary figures**: Figure S1: Sex-stratified age–percentile curves for ventricular volumes. Figure S2: Spearman rank correlations between age and ventricular subcompartment volumes, stratified by sex. Figure S3: CT acquisition characteristics: (A) scanner models and (B) reconstructed slice thicknesses. **Supplementary tables**: Tables S1 and S2: Decade-stratified ventricular volume means and standard deviations in females and males, respectively. Tables S3 and S4: Age-specific ventricular volume percentile reference values in females and males, respectively. Table S5: LLM performance metrics for radiology-report screening, with 95% confidence intervals. Tables S6–S8: Prompts and output schemas for radiology-report analysis and two-step clinical data warehouse screening. Table S9: Comparison with previous ventricular volumetry studies across imaging modalities. **Analysis scripts**: Python scripts for radiology-report LLM evaluation and first- and second-step clinical data warehouse screening.
